# Neutrophil CD64 index as a superior indicator for diagnosing, monitoring bacterial infection, and evaluating antibiotic therapy: a case control study

**DOI:** 10.1186/s12879-022-07725-4

**Published:** 2022-11-28

**Authors:** Yanting Gao, Lihui Lin, Jinyan Zhao, Xia Peng, Li Li

**Affiliations:** Department of Laboratory Medicine, Shanghai General Hospital, Shanghai Jiaotong University, 85 Wujin Road, 200080 Shanghai, China

**Keywords:** Neutrophil CD64 index, Bacterial infection, Antibiotic efficacy

## Abstract

**Background:**

Neutrophil CD64 (nCD64) index has been widely studied as an indication of bacteria-infected diseases, but the exact usage of nCD64 index in monitoring infections remains debated. So this study aims to investigate the functionality of nCD64 index in tracking infections’ progression and evaluating antibiotic therapy.

**Methods:**

160 participants (36 healthy controls, 34 culture-negative patients, 56 respiratory tract infected patients, and 34 bloodstream infected patients) were recruited and divided into groups. Data on nCD64 index, T lymphocyte subsets, and conventional indicators, including white blood cell count, neutrophil to lymphocyte ratio, procalcitonin, and C-reactive protein, were tested and compared.

**Results:**

Bacteria-infected patients had significantly higher nCD64 indexes (*p* < 0.05), especially patients with both bloodstream and respiratory tract infections. The nCD64 index could identify infected patients from culture-negative patients or controls, which conventional indicators cannot achieve. We followed up with 24 infected patients and found that their nCD64 indexes were promptly down-regulated after effective antibiotic therapy (3.16 ± 3.01 vs. 1.20 ± 1.47, *p* < 0.001).

**Conclusion:**

The nCD64 index is a sensitive indicator for clinical diagnosis of bacterial infection, especially in monitoring infection and evaluating antibiotics’ efficacy. Therefore, nCD64 has the potential to improve diagnostic accuracy and provide rapid feedback on monitoring disease progression in infected patients.

**Supplementary Information:**

The online version contains supplementary material available at 10.1186/s12879-022-07725-4.

## Background

Bacterial infection is one of the most common clinical diseases [1]. Since1928 antibiotic therapy has been regarded as the most effective treatment for infections [2]. However, improper and excessive usage of antibiotics has caused a series of problems, such as prolonged hospitalization, increased treatment costs, adverse drug reactions, and antibiotic resistance. The bacterial culture is the most reliable method to separate pathogens, identify the type of pathogens and test bacterial drug resistance. Nevertheless, bacterial cultures take a long time and have low sensitivity, which often result in delay and misdiagnoses.

Meanwhile, the clinical presentations of infection from different pathogens are very similar and confused with inflammatory states (such as trauma, transplant rejection, and vasculitis). Due to this uncertainty, physicians often prescribe empirical antibiotic drugs to patients with suspected bacterial infections as the first course of action. At the same time, there is no ideal indicator that could help quickly determine pathogens and evaluate the efficacy of antibiotic therapy [1]. The most reliable approach remains to be the time-consuming bacterial culture [1]. Over the years, the abuse of antibiotics and the growing antimicrobial resistance have become a vicious cycle which causes high morbidity and mortality [3]. Antibiotic resistance has been named one of the three most important public health threats of the 21st century by the World Health Organization in 2014 [4].

Consequently, a rapid indicator with high sensitivity and specificity is urgently demanded for diagnosing, monitoring, and evaluating bacterial infections. It will lower the risk of antibiotic abuse and reduce antibiotic-associated side effects, mortalities, and treatment failures [1, 5, 6]. Several indicators have been used to identify bacterial infections in clinics, such as white blood cell count (WBC), neutrophil to lymphocyte ratio (NLR), procalcitonin (PCT), and C-reactive protein (CRP). However, they cannot fully meet the needs for diagnostic specificity, sensitive, surveillance, or prognosis evaluation of bacterial infection [7–9].

Recently, the expression of CD64 on peripheral neutrophils, detected via flow cytometry, has been widely used to identify bacteria-infected diseases. CD64, a high-affinity immunoglobulin fragment crystallizable γ receptor I (FcγRI) of human immunoglobulin G, is constitutively expressed on macrophages, monocytes, and eosinophils [10]. In healthy individuals, CD64 expresses on neutrophils at a very low level. However, in bacteria-infected individuals, the expression of CD64 on neutrophils can be markedly elevated (> 10-fold) within a few hours, which allows differentiation between resting and activated neutrophils [11]. Many studies have demonstrated that the expression of CD64 on neutrophils measured as an index had higher sensitivity and specificity for infection: The neutrophil CD64 (nCD64) index significantly increases in bacteria-infected diseases, such as sepsis, systemic infection, bronchitis, and bacterial peritonitis [6, 12].

To date, there is little evidence that the nCD64 index outperforms other serological measures of infection, and only limited studies have reported the association of infection markers with antibiotic efficacy. To this end, we evaluate the value of the nCD64 index in monitoring bacterial infections and antibiotic efficacy. Therefore, this study aims to investigate the accuracy and functionality of the nCD64 index in tracking bacterial infections’ clinical progression and curative effect. To measure the nCD64 index’s performance, we explored the levels of the nCD64 index, WBC, NLR, PCT, CRP, and T lymphocyte subsets among different groups (controls, Negative Bacterial Culture Group, Respiratory Tract Infection Group, and Bloodstream Infection Group), and compared the difference of these indicators in patients before and after receiving effective antibiotic therapy.

## Method and materials

### Study design

The study was conducted from July 2020 to December 2021 at the Shanghai General Hospital, Shanghai Jiaotong University, Shanghai, China. Its design and procedures followed the guidelines of the Helsinki Declaration on human experimentation. The ethics committee of the research institute approved this study, and informed consent forms from participants or their next of kin had been obtained prior to their inclusions in the study.

### Case and control recruitment

**Fig. 1:**
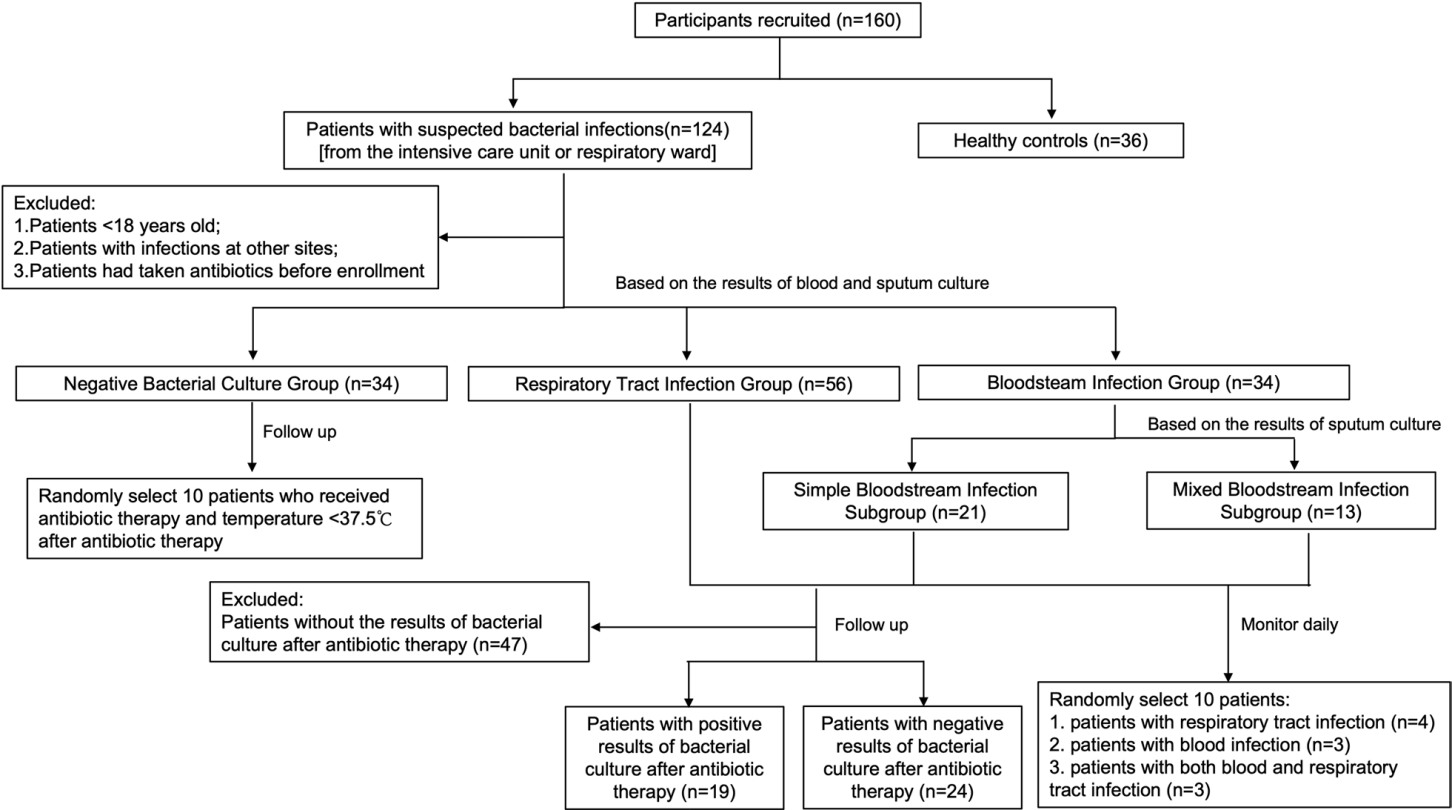
Study flowchart. Representation of each step of the study, recruited participants included healthy controls and patients with suspected bacterial infections. Patients with suspected bacterial infections were divided based on bacterial culture results, including culture-negative patients, respiratory tract infected patients, and bloodstream infected patients. The bloodstream infected patients were further divided into Simple Bloodstream Infection Subgroup and Mixed Bloodstream Infection Subgroup, based on sputum culture results. All infected patients and 10 patients with negative bacterial cultures were followed up after receiving antibiotic therapy

Figure 1 shows the study flowchart. 36 healthy controls (age 32–95 years) were recruited from subjects who participated in annual health screenings at the Shanghai General Hospital, Shanghai Jiaotong University. 124 inpatients (age 20–97 years) with suspected respiratory tract infections or bloodstream infections from the intensive care unit (ICU) or respiratory ward were recruited. The suspected infection was defined as inpatients with one or several clinical symptoms (fever, chill, cough, sneeze, tachypnea, respiratory distress, chest wheezing), inflammatory markers and microbiological cultures were requested by attending physicians, and empirical antibiotic therapy was initiated [13]. We excluded patients: (1) age < 18 years old, (2) with infections at other sites besides respiratory tract infections and bloodstream infections, and (3) who had taken antibiotics before enrollment. All enrolled patients were classified into three groups based on the results of blood or sputum cultures:

1. Negative Bacterial Culture Group (n = 34): patients with no evidence of infection by negative blood and sputum cultures. This group includes some patients with no infection and others having bacterial infections but with negative bacterial culture results.
2. Respiratory Tract Infection Group (n = 56): patients with respiratory tract infection proven by positive sputum culture and negative blood culture.
3. Bloodstream Infection Group (n = 34): patients with bloodstream infection proven by positive blood culture.

The Bloodstream Infection Group was further divided into two subgroups based on the results of sputum cultures: (1) Simple Bloodstream Infection Subgroup: patients with bloodstream infection only proven by positive blood culture and negative sputum culture (n = 21); (2) Mixed Bloodstream Infection Subgroup: patients with both bloodstream and respiratory tract infection proven by positive blood and sputum cultures (n = 13).

In addition, we followed up with the infected patients above and recorded their bacterial culture results after administering antibiotic therapy. During the study period, we recorded a total of 24 infected patients (age 23–83 years) whose blood and sputum cultures turned negative and had no clinical symptoms of infection after effective antibiotic therapy. Amongst these 24 patients, 15 were from the Respiratory Tract Infection Group, 6 were from the Simple Bloodstream Infection Subgroup, and 3 were from the Mixed Bloodstream Infection Subgroup. At the same time, we also recorded 19 infected patients (age 23–95 years) whose blood and sputum cultures remained positive after antibiotic therapy. Amongst these 19 patients, 11 were from the Respiratory Tract Infection Group, 3 were from the Simple Bloodstream Infection Subgroup, and 5 were from the Mixed Bloodstream Infection Subgroup. Moreover, we followed up with 10 patients randomly from the Negative Bacterial Culture Group who received antibiotic therapy and had no clinical symptoms of infection after antibiotic therapy, and recorded the changes in their indicators before and after antibiotic therapy. We also randomly selected 10 infected patients and monitored their nCD64 index, PCT, and temperature changes daily. Amongst these 10 patients, 4 were from the Respiratory Tract Infection Group, 3 were from the Simple Bloodstream Infection Subgroup, and 3 were from the Mixed Bloodstream Infection Subgroup.

### Samples collection

Patients’ peripheral blood samples, blood cultures, and sputum cultures were collected simultaneously and examined before antibiotic therapy. Sterile blood culture bottles (BD BACTEC™ Plus Aerobic/F and BD BACTEC™ Lytic/10 Anaerobic/F) containing 10 mL of fresh blood were incubated in the automated BD BACTEC™ FX system at 37ºC for 7 days, after which it was considered negative. The blood culture, sputum culture, and the identification of pathogens were performed by the Clinical Microbiology Laboratory of the Department of Laboratory Medicine. The distribution of bacteria strains in patients is presented in Additional file 3: Table S1.

### Measurement of conventional indicators

We used the Mindray BC-5390 hematology analyzer (Mindray Bio-Medical Electronics Co., Ltd., Shenzhen, China) and corresponding diagnostic kits to measure routine blood counts and CRP. The WBC and NLR were calculated based on routine blood counts. We measured serum PCT levels by electro-chemiluminescence immunoassay in Roche Cobas e601 automatic analyzer with the Elecsys BRAHMS PCT assay reagent (Roche Diagnostics GmbH, Mannheim, Germany). All operations were strictly conducted according to the instructions provided by the instrument and reagent manufacturers.

### Measurement of nCD64 index and T lymphocyte subsets via flow cytometry

**Fig. 2:**
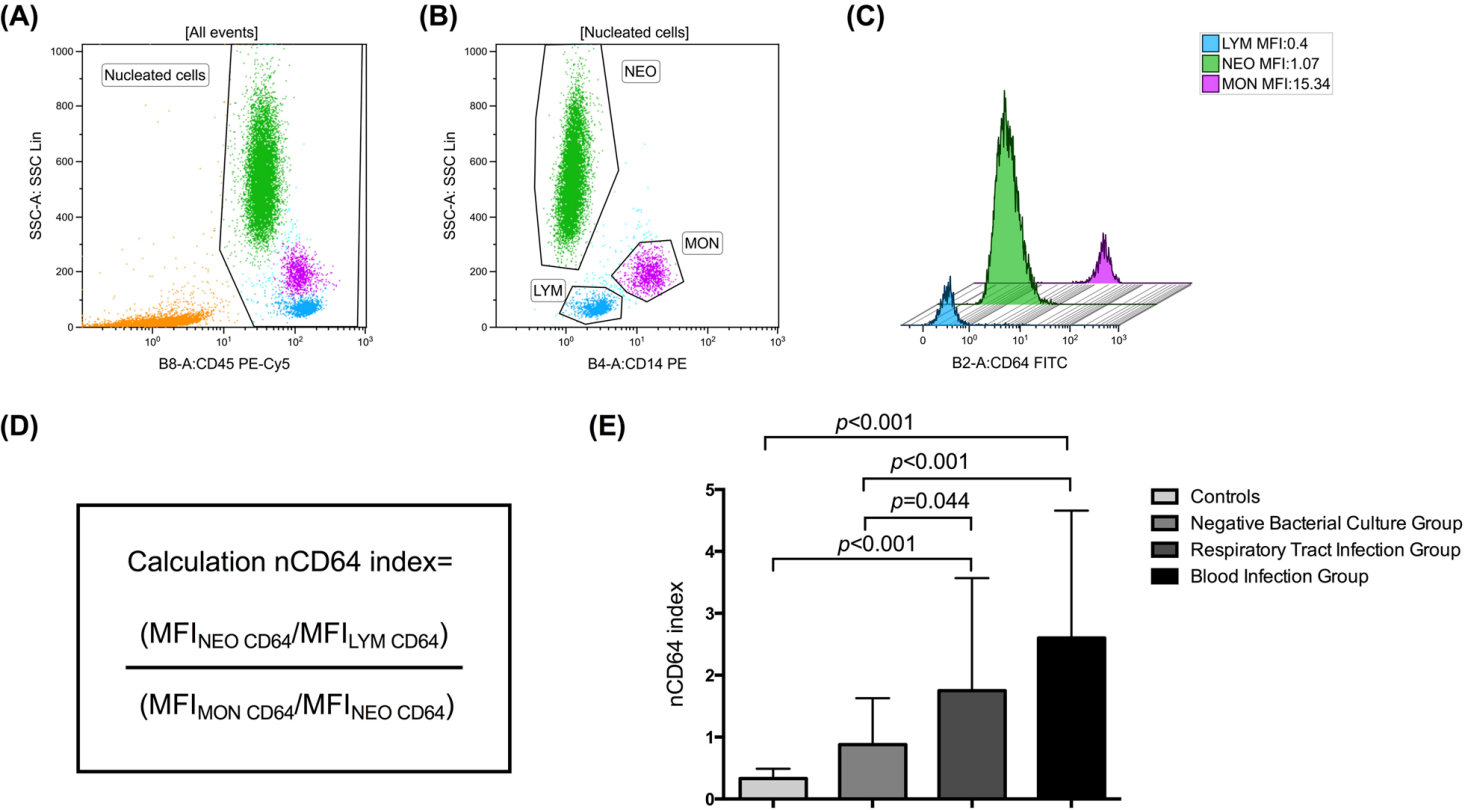
Comparison of the nCD64 index among groups. **A**–**D** The differential expression of CD64 on leucocyte surface in a representative bacteria-infected patient. **A** Nucleated cells were gated according to SSC and pan-leucocyte marker CD45 distribution; **B** Lymphocytes (blue, LYM gate), neutrophils (green, NEO gate), and monocytes (purple, MON gate) were gated from nucleated cells according to CD14 distribution; **C** The histogram of CD64 expression (MFI) on neutrophils, lymphocytes, and monocytes; **D** The formula of nCD64 index calculation. **E** The nCD64 levels are significantly higher in Respiratory Tract Infection Group and Bloodstream Infection Group than in controls or Negative Bacterial Culture Group, respectively (n = 36 for controls; n = 34 for Negative Bacterial Culture Group; n = 56 for Respiratory Tract Infection Group; n = 34 for Bloodstream Infection Group). *NEO* neutrophils, *LYM* lymphocytes, *MON* monocytes, *SSC*  side scatter, *MFI* mean fluorescence intensity, *FITC *fluorescein isothiocyanate, *PE * phycoerythrin, *PE-Cy5* phycoerythrin-cyanine5, *nCD64 index* neutrophil CD64 index. Data in **B** were analyzed by the one-way ANOVA with Bonferroni multiple comparison tests

Whole peripheral blood samples were stained with anti-human CD64 fluorescein isothiocyanate (CD64-FITC, Beckman Coulter, USA), anti-human CD14 phycoerythrin (CD14-PE, Beckman Coulter, USA), and anti-human CD45 phycoerythrin-cyanine5 (CD45-PE-Cy5, Beckman Coulter, USA). After staining, red blood cells were lysed with Erythrocyte Lysis Buffer (BD Biosciences, USA) to obtain peripheral blood nucleated cells. We used full-spectrum flow cytometry Cytek Northern Lights-CLC (NL-CLC, Cytekbios, China) to examine nucleated cells stained with fluorescent. The scatter plot was drawn with side scatter (SSC) and pan-leucocyte marker CD45 to gate nucleated cells (Fig. 2 A). Then, neutrophils (NEO), lymphocytes (LYM), and monocytes (MON) were gated from nucleated cells according to CD14 distribution (Fig. 2B). The mean fluorescence intensity (MFI) of CD64 in neutrophils (NEO), lymphocytes (LYM), and monocytes (MON) were acquired, respectively (Fig. 2 C). The nCD64 index was calculated with the following formula: nCD64 index= (MFI_NEO CD64_/MFI_LYM CD64_)/ (MFI_MON CD64_/MFI_NEO CD64_) (Fig. 2D). In the calculation, the MFI of CD64 on LYM and MON served as the internal negative and positive controls.

T lymphocyte subsets were measured with a similar approach. We stained whole peripheral blood samples with anti-human CD3 fluorescein isothiocyanate (CD3-FITC, Beckman Coulter, USA), anti-human CD4 phycoerythrin (CD4-PE, Beckman Coulter, USA), anti-human CD8 phycoerythrin-cyanine5 (CD8-PE-Cy5, Beckman Coulter, USA), and anti-human CD45 phycoerythrin-cyanine7 (CD45-PE-Cy7, Beckman Coulter, USA). Afterwards, red blood cells were lysed with Erythrocyte Lysis Buffer (BD Biosciences, USA) to obtain peripheral blood nucleated cells. We detected nucleated cells stained with fluorescent via flow cytometry (NL-CLC, Cytekbios, China) and calculated counts of CD3^+^ T cell, CD4^+^ T cell, and CD8^+^ T cell using the volume method.

### Statistical analysis

Data were analyzed using SPSS for Windows version 24.0 software (SPSS, Chicago, IL, USA). We calculated numbers and proportions for qualitative data and presented results as the mean ± standard deviation (SD) for quantitative data. The Kolmogorov-Smirnoff test assessed the normality of the data. We then used paired t-test, independent Student’s t-test, and one-way ANOVA followed by Bonferroni multiple comparison tests to assess normal distribution data. In addition, to assess non-normal distribution data and qualitative data, we used the χ2 test, the Mann-Whitney U test, Wilcoxon signed ranks test, and Kruskal-Wallis followed by Bonferroni multiple comparison tests. The stepwise multivariable logistic regression analysis was performed to identify the association between the nCD64 index and bacterial infections. And receiver operating characteristic (ROC) curves were plotted to explore the significance of PCT and nCD64 index for differentiating infected patients from culture-negative patients. We calculated the predicted probabilities of combining PCT and nCD64 index through logistic regression analysis and plotted ROC curves based on the predicted probabilities. At the same time, we calculated and compared the areas under the curve (AUC), the cutoff value, the sensitivity and the specificity of different indicators. In all cases, differences were statistically significant with *p* < 0.05.

## Results

### Clinical and laboratory characteristics of participants according to groups

A total of 160 participants were involved in this study, including 36 healthy controls, 34 patients with negative bacterial cultures, 56 patients with respiratory tract infections, and 34 patients with bloodstream infections. There was no significant difference in age and gender among them. Table 1 shows the clinical and laboratory characteristics of all participants.

**Table 1:** Clinical and laboratory characteristics of participants

	Healthy controls (n = 36)	Negative Bacterial Culture Group (n = 34)	Respiratory Tract Infection Group (n = 56)	Bloodstream Infection Group (n = 34)	*p* value
Demographic parameters					
Age, years	61.89 ± 16.46	63.59 ± 13.82	69.86 ± 16.51	64.76 ± 15.71	0.087
Male, n(%)	25 (69.44%)	18 (52.94%)	42 (75.00%)	25 (73.53%)	0.148
Temperature (℃)	/	37.57 ± 0.77	37.71 ± 0.69	38.19 ± 0.84	0.002^d,e^
Sputum culture					
Positive, n(%)	/	0 (0)	56 (100.00%)	13 (38.24%)	
Negative, n(%)	/	34 (100.00%)	0	21 (61.76%)	
Blood culture					
Positive, n(%)	/	0 (0)	0 (0)	34 (100.00%)	
Negative, n(%)	/	34 (100.00%)	56 (100.00%)	0 (0)	
Laboratory indicators					
WBC, ×10^9^/L	6.57 ± 1.32	9.61 ± 4.91	10.33 ± 4.40	10.28 ± 4.80	< 0.001^a,b,c^
NLR, %	1.81 ± 0.37	8.67 ± 6.22	15.63 ± 20.13	39.40 ± 81.00	< 0.001^a,b,c^
PCT, ng/mL	0.03 ± 0.01	0.36 ± 0.47	1.82 ± 4.29	4.98 ± 10.00	< 0.001^a,b,c,d^
CRP, mg/L	0.92 ± 0.95	56.16 ± 59.10	93.81 ± 85.38	100.83 ± 73.27	< 0.001^a,b,c^
CD3^+^ T cell count, ×10^6^/L	1298.06 ± 447.89	675.85 ± 331.00	771.98 ± 879.89	495.24 ± 354.65	< 0.001^a,b,c^
CD4^+^ T cell count, ×10^6^/L	692.75 ± 220.20	409.53 ± 216.74	380.59 ± 394.26	254.27 ± 171.14	< 0.001^a,b,c^
CD8^+^ T cell count, ×10^6^/L	490.14 ± 191.93	215.82 ± 140.46	341.59 ± 511.73	205.27 ± 219.94	0.001^a,c^

Data were expressed as mean ± standard deviation (SD) or n(%). The One-way ANOVA followed by Bonferroni multiple comparison tests, the Kruskal-Wallis followed by Bonferroni multiple comparison tests, or χ2 test were used

^a^*p*<0.05 for the difference between the controls and Negative Bacterial Bulture Group

^b^*p*<0.05 for the difference between the controls and Respiratory Tract Infection Group

^c^*p*<0.05 for the difference between the controls and Bloodstream Infection Group

^d^*p*<0.05 for the difference between the Negative Bacterial Culture Group and Bloodstream Infection Group

^e^*p*<0.05 for the difference between the Respiratory Tract Infection Group and Bloodstream Infection Group

*WBC* white blood cell count, *NLR* neutrophil to lymphocyte ratio, *PCT* procalcitonin, *CRP* C-reactive protein

We compared peripheral blood conventional indicators (WBC, NLR, PCT, CRP) and T lymphocyte subsets among groups to evaluate indicators’ diagnostic power in bacterial infections. Compared to controls, WBC, NLR, PCT, and CRP were significantly higher, while the counts of CD3 + T cell and CD4 + T cell were significantly lower in culture-negative patients and infected patients (p < 0.05); in additon, CD8 + T cell counts were significantly lower in culture-negative patients and bloodstream-infected patients (p < 0.05). Compared to the Negative Bacterial Culture Group, only the PCT level was significantly elevated in the Bloodstream Infection Group (p = 0.002). It supported earlier claims that conventional indicators and T lymphocyte counts lack specificity in indicating bacterial infections. These infection markers can help clinical practitioners identify which patients might have suspected infections, but cannot confirm which patients have bacterial infections. Therefore, more specific clinical indicators are required to confirm infection accurately.

### nCD64 index specifically increased in infected patients

To evaluate the effectiveness of the nCD64 index in identifying bacterial infections, we compared the levels of the nCD64 indexes among different groups (Fig. 2E). The nCD64 indexes in infected patients (including respiratory tract infected patients and bloodstream infected patients) were significantly higher than that in controls (1.75 ± 1.82 vs. 0.33 ± 0.16, *p* < 0.001; 2.60 ± 2.06 vs. 0.33 ± 0.16, *p* < 0.001) and culture-negative patients (1.75 ± 1.82 vs. 0.88 ± 0.75, *p* = 0.044; 2.60 ± 2.06 vs. 0.88 ± 0.75, *p* < 0.001). The levels of the nCD64 indexes between controls and culture-negative patients had no statistically significant differences (0.33 ± 0.16 vs. 0.88 ± 0.75, *p* = 0.725). Thus it signals that the nCD64 index could be used as an indicator specifically for distinguish bacteria-infected paitents from healthy controls and culture-negative patients.

### Elevated nCD64 index significantly associated with bacterial infections

To further confirm the high nCD64 index could indicate bacterial infections, we used stepwise multivariable logistic regression analysis to show the association between the nCD64 index and infections in respiratory tract infected or bloodstream infected patients compared to culture-negative patients. The results revealed that elevated nCD64 indexes were significantly associated with respiratory tract infection and bloodstream infections after adjusting for age, gender, temperature, WBC, NLR, CRP, and the counts of T lymphocyte subsets (Table 2).

**Table 2:** Logistic regression analysis of the association of nCD64 and PCT with bacterial infections in respiratory tract infected patients and bloodstream infected patients, compared to culture-negative patients

	*p* value	OR	95% CI
Respiratory Tract Infection Group			
nCD64 index	0.019	1.998	1.122–3.556
PCT (ng/mL)	0.125	1.854	0.842–4.081
Bloodstream Infection Group			
nCD64 index	0.033	2.390	1.072–5.328
PCT (ng/mL)	0.086	2.550	0.875–7.434

Stepwise multivariable logistic regression analysis was used

*nCD64 index* neutrophil CD64 index, *PCT* procalcitonin, *OR* odds ratio, *CI* confidence interval

### Higher nCD64 index, NLR, and lower T lymphocyte counts in patients with both bloodstream and respiratory tract infection

To further our understanding of the value of the nCD64 index in monitoring complicated bacterial infections, we divided the 34 bloodstream-infected patients into two subgroups: Simple Bloodstream Infection Subgroup (n = 21, 16 males and 5 females, 63.48 ± 18.86 years) and Mixed Bloodstream Infection Subgroup (n = 13, 9 males and 4 females, 66.85 ± 8.84 years). Then we compared the differences in the nCD64 indexes, conventional indicators (WBC, NLR, PCT, CRP), and T lymphocyte subsets among Respiratory Tract Infection Group, Simple Bloodstream Infection Subgroup, and Mixed Bloodstream Infection Subgroup. There were also no significant differences in age and gender among groups (*p* = 0.304, *p* = 0.895, respectively).

**Fig. 3:**
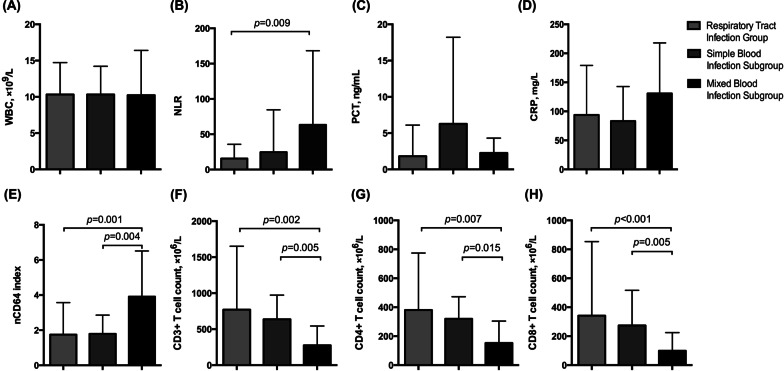
Comparison of indicators among groups. **A**–**H** The levels of WBC, NLR, PTC, CRP, nCD64 index, and T lymphocyte subsets (including CD3 + T cell, CD4 + T cell, and CD8 + T cell) among groups. Mixed Bloodstream Infection Subgroup has significantly higher nCD64 index and NLR levels, whereas it has significantly lower counts of CD3 + T cells, CD4 + T cells, and CD8 + T cells. *WBC *white blood cell count; *NLR* neutrophil to lymphocyte ratio, *PCT* procalcitonin, *CRP* C-reactive protein, *nCD64 index*  neutrophil CD64 index. Data in **A**–**H** were analyzed by independent Student’s t-test or Mann-Whitney U test

As shown in Fig. 3, the nCD64 indexes were significantly higher, as well as the counts of CD3^+^ T cell, CD4^+^ T cell, and CD8^+^ T cell were significantly lower in the Mixed Bloodstream Infection Subgroup, compared to the Respiratory Tract Infection Group and Simple Bloodstream Infection Subgroup (p < 0.05). Meanwhile, the NLR was significantly higher in Mixed Bloodstream Infection Subgroup than in the Respiratory Tract Infection Group (63.34 ± 105.03 vs. 15.63 ± 20.13, *p* = 0.009). However, other conventional indicators had no significant differences among groups (*p* > 0.05). Therefore, the nCD64 index, NLR, and T lymphocyte subsets may have the potential to indicate complicated bacterial infections.

### Similar diagnostic value of PCT and nCD64 index in differentiating patients with bacterial infections from patients with negative bacterial cultures via ROC curves

**Fig. 4:**
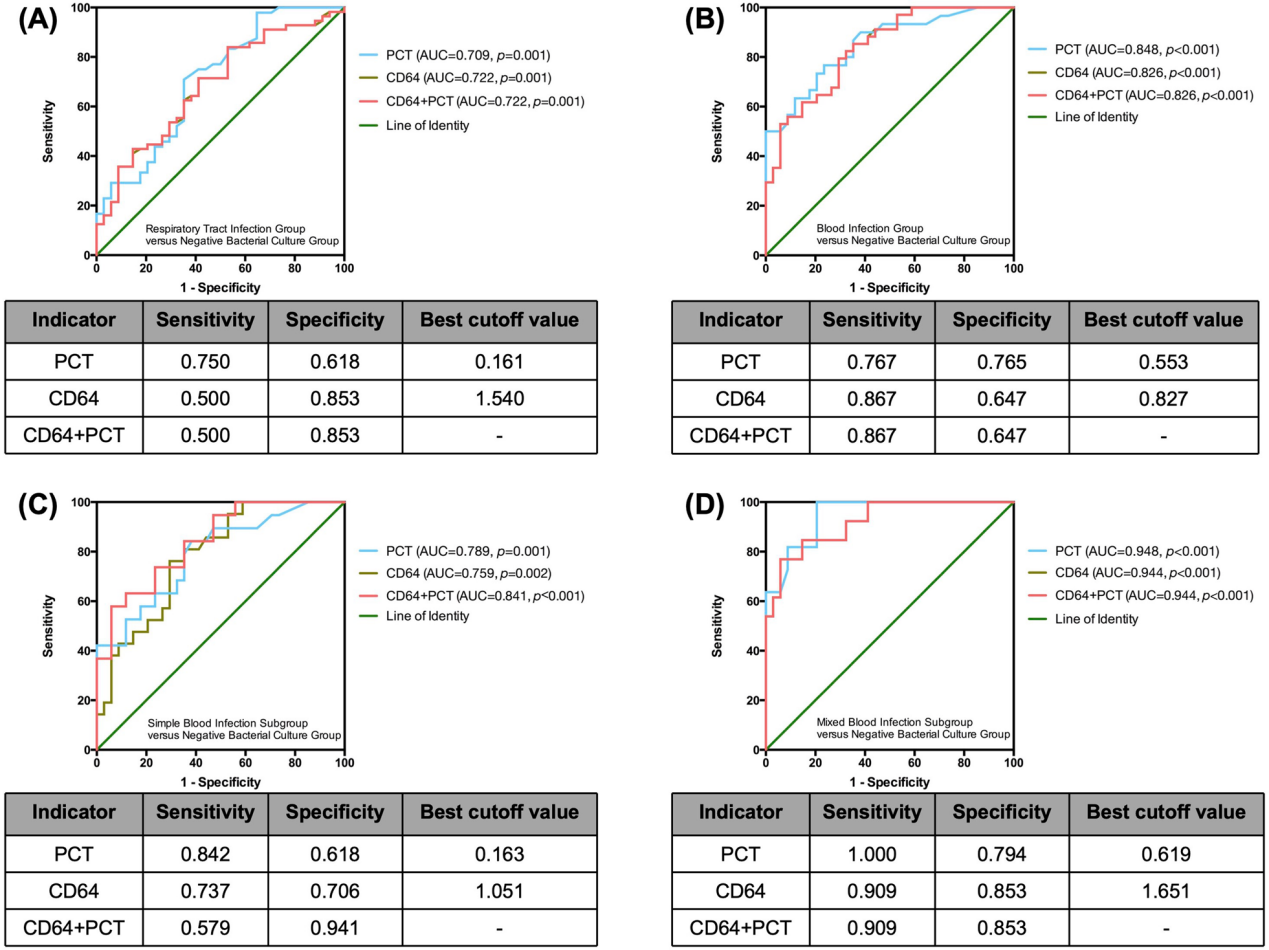
ROC curves of PCT, nCD64 index and their combination. **A** ROC curves for differentiating the Respiratory Tract Infection Group from the Negative Bacterial Culture Group; **B** ROC curves for differentiating the Bloodstream Infection Group from the Negative Bacterial Culture Group; **C** ROC curves for differentiating the Simple Bloodstream Infection Subgroup from the Negative Bacterial Culture Group; **D** ROC curves for differentiating the Mixed Bloodstream Infection Subgroup from the Negative Bacterial Culture Group. *ROC* receiver operating characteristic, *AUC* areas under ROC curves, *PCT* procalcitonin, *nCD64 index* neutrophil CD64 index

PCT is commonly used to help diagnose bacterial infections. To estimate whether the diagnostic value of the nCD64 index was superior to that of PCT, we analyzed the ROC curves of PCT, nCD64 index, and their combination (Fig. 4). The AUC of PCT and nCD64 index to distinguish infected patients from culture-negative patients was similar. The combination of PCT and nCD64 index did not improve the diagnostic value. It implied that the diagnostic value of the nCD64 index was not better than that of PCT.

### nCD64 indexes significantly decreased in infected patients after receiving effective antibiotic therapy

**Fig. 5:**
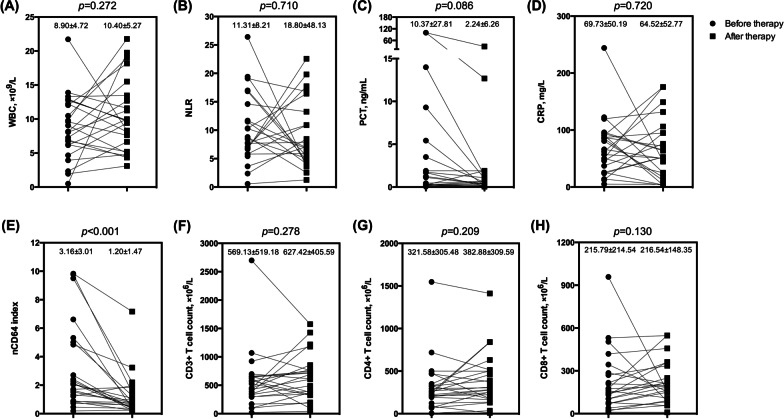
Comparison of indicators in infected patients whose cultures turned negative after effective antibiotic therapy. *WBC* white blood cell count, *NLR *neutrophil to lymphocyte ratio, *PCT* procalcitonin, *CRP* C-reactive protein, *nCD64 index* neutrophil CD64 index. Data in **A**–**H** were analyzed by the paired t-test or Wilcoxon signed ranks test, and expressed as mean ± SD

**Fig. 6:**
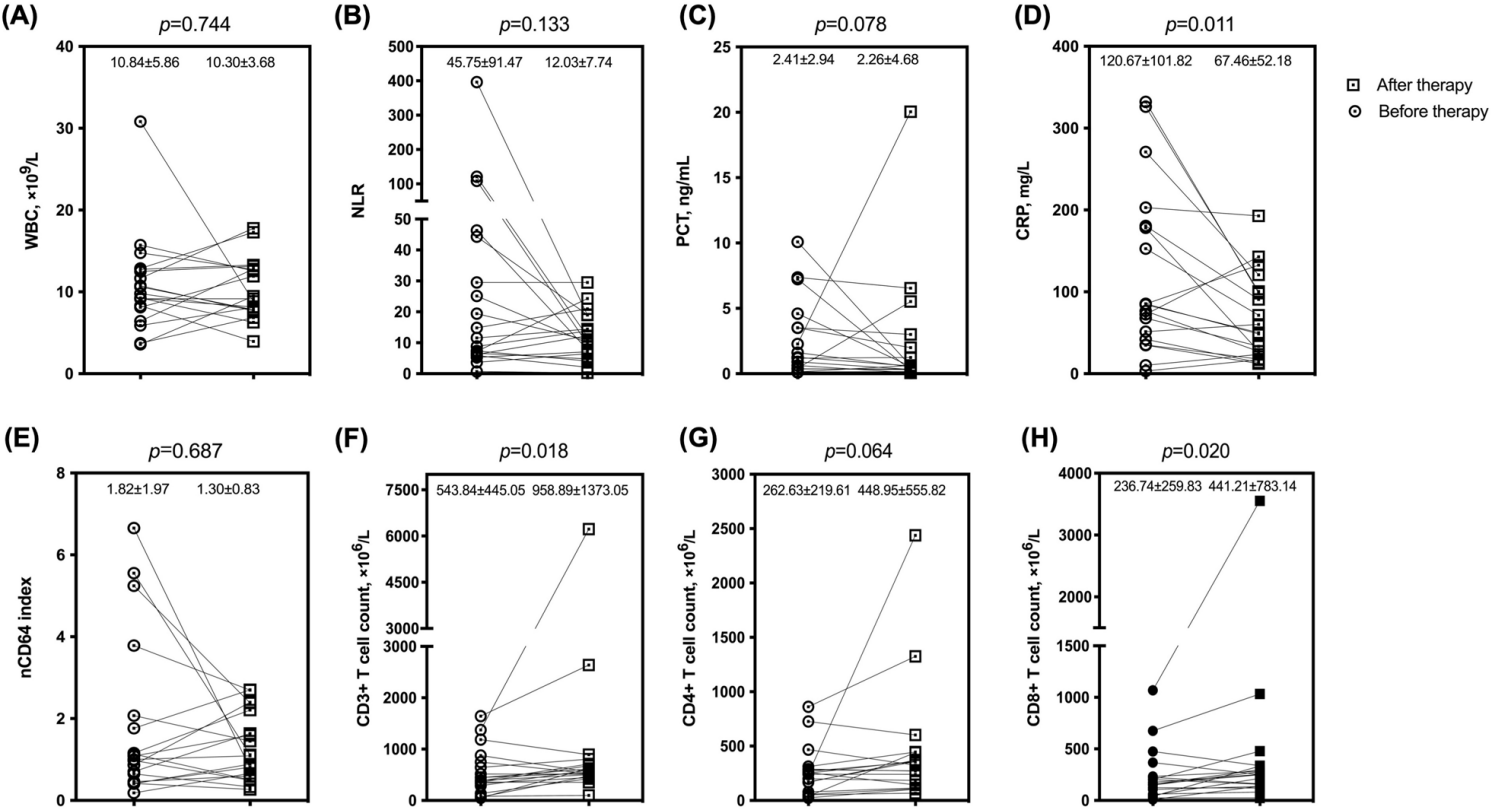
Comparison of indicators in infected patients whose cultures remained positive after antibiotic therapy. *WBC* white blood cell count, *NLR *neutrophil to lymphocyte ratio, *PCT* procalcitonin, *CRP* C-reactive protein, *nCD64 index* neutrophil CD64 index. Data in (**A**–**H**) were analyzed by the paired t-test or Wilcoxon signed ranks test, and expressed as mean ± SD

We acknowledge that it is essential to seek an ideal indicator to evaluate antibiotic efficacy and guide antibiotic therapy. This section of the study aimed to test the effectiveness of nCD64 index as a potential candidate. During the study period, we followed up and collected 24 infected patients (18 males and 6 females, 62.96 ± 14.61 years) whose bacterial cultures returned negative after antibiotic treatment. Patients’ laboratory indicators were compared before and after antibiotic therapy (Fig. 5). The decline of the nCD64 index in patients after receiving effective antimicrobial treatment was significant (*p* < 0.001), while the alteration of other indicators was not noticeable. At the same time, we also observed the levels of nCD64 indexes in 19 infected patients (15 males and 4 females, 67.95 ± 17.37 years) whose bacterial cultures remained positive after antibiotic therapy (Fig. 6). And we found that nCD64 indexes in these patients had no significant difference before and after antibiotic therapy (*p >* 0.05). So the significant decline in the nCD64 index might indicate that antibiotic therapy is effective.

Meanwhile, we followed up with 10 randomly selected patients from the Negative Bacterial Culture Group who received antibiotic therapy and observed that there was no difference in the nCD64 index in these patients before and after antibiotic therapy (Additional file 1: Fig. S1). We also randomly selected 10 infected patients and monitored the changes in the nCD64 index, PCT, and temperature in these patients daily since they received antibiotic therapy. We found that the downward tendency of the nCD64 index was closely related to antibiotic efficacy and prognosis (Additional file 2: Fig. S2). Thus, it vehemently claimed that the nCD64 index is an excellent indicator for monitoring bacterial infection and evaluating the efficacy of antibiotic therapy.

## Discussion

With the increasing antimicrobial resistance, bacterial infections are becoming a severe problem that cannot be neglected. Due to the time-consuming nature and low sensitivity results of bacterial culture, rapid indicators that could help identify bacterial infections and guide antibiotic therapy are urgently needed. Hence, this study focused on the novel indicator nCD64 index in bacterial infections and explored the value of the nCD64 index in the diagnosis, surveillance, and prognosis evaluation of bacterial infections. In this study, we found that the level of the nCD64 index significantly increased in bacteria-infected patients compared to controls and culture-negative patients, and the elevated nCD64 index was significantly associated with bacterial infections. Meanwhile, there was no significant difference in the nCD64 indexes between controls and culture-negative patients. Thus, the nCD64 index could be a specific indicator to indicate bacterial infections. However, the diagnostic effectiveness of the nCD64 index as an identifier for differentiating bacteria-infected patients from culture-negative patients was similar to that of PCT. In terms of tracking bacteria-infected clinical progression, the nCD64 index was superior to PCT. When infected patients received effective antibiotic therapy, the high nCD64 index was promptly and largely downregulated, while other indicators had no significant alteration. Conversely, we also confirmed that when infected patients received ineffective antibiotic therapy, the nCD64 index remained high. Therefore, we speculated that the nCD64 index is a superior indicator in monitoring bacterial infections and evaluating the efficacy of antibiotic therapy than conventional indicators, such as WBC, NLR, CRP, and PCT.

Fever is a primary clinical symptom for early recognition of infection, but its diagnostic accuracy is very low. Various factors (such as primary diseases, inflammatory responses, and central lesions) can result in fevers. Therefore, other infection indicators are necessary to assist in monitoring clinical infections. Conventional indicators include WBC, NLR, CRP, PCT. WBC and NLR are straightforwardly calculated from routine blood examination, and extensive studies have shown that WBC and NLR significantly increase in bacteria-infected diseases. However, the specificity and accuracy of WBC and NLR in distinguishing infectious diseases from noninfectious inflammatory disorders are very low [7]. This study also verified that levels of WBC and NLR in infected patients were significantly higher, but they could not identify bacteria-infected patients from culture-negative patients.

CRP is another common indicator for infection diseases. It is a well-studied acute phase protein produced by the liver and increases within the first 24–48 h of bacterial infections [14]. Patients with bacterial infections commonly present significantly higher CRP levels than those without [15, 16]. However, CRP is not a specific marker; many causes could increase CRP levels, such as autoimmune disease, cancer surgery, and trauma [17]. In addition to CRP, PCT is also commonly used in clinical practices as an infection indicator. It is an acute-phase protein, mainly synthesized and secreted by thyroid follicular cells. Under physiological conditions, serum PCT concentration is negligible; once bacterial infections occur, PCT increases within the first 3–4 h, peaks at 6–8 h, and lasts for at least 24 h [18]. Although several noninfectious conditions (trauma, surgery, and carcinoma) could also increase PCT levels, PCT still has better accuracy in differentiating bacterial infections than CRP, WBC, and NLR [8, 9]. Studies have reported that the serum PCT level significantly increases in patients with bacterial infections, while the level moderately increases in negative bacterial culture conditions [8]. It is similar to the results of this study. Several meta-analyses indicated that PCT-guided therapy significantly reduced antibiotic exposure [19, 20], and serum PCT concentration declined quickly during the antibiotic treatment. Nevertheless, the decline of PCT levels after antibiotic treatment was not as quick as nCD64 indexes in this study. Thus, we speculated that the nCD64 index might be better than PCT in monitoring antibiotic therapy.

nCD64, an indicator of bacteria-infected diseases, has been widely discussed in various studies. The diagnostic potential of nCD64 was first proposed in 1995 [21], and studies have since confirmed that nCD64 significantly increased in patients with systemic infection and sepsis [21]. In 2008, two studies first demonstrated that nCD64 as an index had high sensitivity and specificity for measuring infection when used among neonatal intensive care unit patients [22, 23]. A series of studies reported that the nCD64 index could be used as a sensitive and specific indicator for diagnosing spontaneous bacterial peritonitis in cirrhotic patients [24]; the nCD64 index was helpful for the early identification of bacterial infections in febrile patients in the hematology department [25]; and the nCD64 index could be used to diagnose sepsis and predict sepsis in critically ill patients [26–28]. Our data also proved that the nCD64 index could identify bacteria-infected patients from culture-negative patients and controls. Meanwhile, the levels of the nCD64 indexes in patients with both bloodstream and respiratory tract infections were significantly higher, indicating that the nCD64 index could also be used to predict complicated multi-site infections. The expression of CD64 on neutrophils strongly upregulates within 4–6 h stimulated by cytokines such as interferon-gamma (IFN-γ) and granulocyte colony-stimulating factor (G-CSF), when infections occur. Once infections are eradicated, experimentally represented by the removal of IFN in vivo, nCD64 levels drop radically within 48 h [29–31]. Thus, the nCD64 index could be an ideal indicator to direct antibiotic therapy for infections. Aikaterini Dimoula et al. reported that septic patients receiving inappropriate empirical antibiotics had persistently elevated nCD64 expression, whereas the expression decreased over time in patients receiving appropriate antibiotics [32]. Another study reported that the nCD64 indexes significantly decreased in patients with mycobacterium tuberculosis infection after anti-tuberculosis therapy [33]. In this study, the high nCD64 index was markedly reduced in infected patients receiving effective antibiotic treatment, and the decline of the nCD64 index was related to antibiotic efficacy and prognosis. Therefore, the nCD64 index has the potential to monitor and evaluate the efficacy of antibiotic therapy for clinics.

Considering the vital function of T lymphocytes in anti-infective immunity as an identifier of the pathogen and eradicating infection [34], T lymphocyte subsets are widely measured and used in the clinic. Increasing studies have paid attention to the alteration of T lymphocyte subsets in bacteria-infected diseases, particularly in sepsis, and reported that the absolute counts of T lymphocytes (including CD3^+^ T cell, CD4^+^ T cell, and CD8^+^ T cell) significantly decreased in severe sepsis patients [35, 36]. We also showed that the counts of CD3^+^ T cell, CD4^+^ T cell, and CD8^+^ T cell in infected patients were significantly lower than in controls, especially in patients with both bloodstream and respiratory tract infections. Therefore, we concluded that T lymphocyte subsets (CD3^+^ T cell, CD4^+^ T cell, and CD8^+^ T cell) could also be used to help monitor infection and assess the severity of bacterial infection.

We acknowledge that this study has some limitations. The number of bacteria-infected patients enrolled in the study is limited, especially the number of bloodstream-infected cases. A prospective study of more cases should be conducted in the future. Secondly, for analyzing the value of the nCD64 index in evaluating antibiotic efficacy, except for infected patients who were measured both before and after antibiotic therapy, we only monitored 10 infected patients daily during the antibiotic therapy period. For these reasons, the representativeness of these results may be limited. Thirdly, for patients recruited in this study from ICU or respiratory wards, the heterogenicity of patients, and the severity of infections could affect the results. So based on the results of this study, it might be more significant to focus on patients with a single pathogen infection from the same ward and further explore the performance of the nCD64 index in bacterial infection. It would be worth clarifying the difference of the nCD64 index in patients with different degrees of bacterial infections through more detailed research. Last but not least, in this study, patients in the Negative Bacterial Culture Group included patients with no infection and patients having bacterial infections but with negative bacterial culture results. It would be of more clinical significance to identify infected patients from culture-negative patients by using other detection methods such as metagenomic, and further explore the association between nCD64 indexes and bacterial infections in the future.

In conclusion, although the diagnostic value of the nCD64 index and PCT for bacterial infections is similar, the nCD64 index is still a superior indicator in monitoring infections and evaluating the efficacy of antibiotic therapy. The nCD64 index has excellent potential to improve clinical diagnostic accuracy and provide rapid feedback in monitoring disease progression in bacteria-infected patients.

## Supplementary Information

**Additional file 1: Figure S1.** Comparison of indicators in patients with negative bacterial culture beforeand after appropriate antibiotic therapy.

**Additional file 2: Figure S2.** The daily alteration of the nCD64 index, PCT, and temperature in infectedpatients.

**Additional file 3: Table S1.** The distributionof bacteria strains.

## Data Availability

The datasets used and/or analyzed during the current study are available from the corresponding author on reasonable request.

## References

[1] Deusenbery C, Wang Y, Shukla A (2021). Recent innovations in bacterial infection detection and treatment. ACS Infect Dis.

[2] Hutchings MI, Truman AW, Wilkinson B (2019). Antibiotics: past, present and future. Curr Opin Microbiol.

[3] Akova M (2016). Epidemiology of antimicrobial resistance in bloodstream infections. Virulence.

[4] World Health Organization (2014). Antimicrobial resistance: global report on surveillance.

[5] de Jong E, van Oers JA, Beishuizen A, Vos P, Vermeijden WJ, Haas LE, Loef BG, Dormans T, van Melsen GC, Kluiters YC (2016). Efficacy and safety of procalcitonin guidance in reducing the duration of antibiotic treatment in critically ill patients: a randomised, controlled, open-label trial. Lancet Infect Dis.

[6] Cong S, Ma T, Di X, Tian C, Zhao M, Wang K: Diagnostic value of neutrophil CD64, procalcitonin, and interleukin-6 in sepsis: a meta-analysis. Meta-Analysis 2021, 21(1):384. 10.1186/s12879-021-06064-0 PMC8072745 33902476

[7] ten Oever J, Netea MG, Kullberg BJ (2016). Utility of immune response-derived biomarkers in the differential diagnosis of inflammatory disorders. J Infect.

[8] Tang JH, Gao DP, Zou PF (2018). Comparison of serum PCT and CRP levels in patients infected by different pathogenic microorganisms: a systematic review and meta-analysis. Braz J Med Bio Res.

[9] Memar MY, Varshochi M, Shokouhi B, Asgharzadeh M, Kafil HS (2017). Procalcitonin: The marker of pediatric bacterial infection. Biomed Pharmacother.

[10] Hu BQ, Yang Y, Zhao CJ, Liu DF, Kuang F, Zhang LJ, Yu X (2019). Accuracy of neutrophil CD64 expression in diagnosing infection in patients with autoimmune diseases: a meta-analysis. Clin Rheumatol.

[11] Mortaz E, Alipoor SD, Adcock IM, Mumby S, Koenderman L (2018). Update on neutrophil function in severe inflammation. Front Immunol.

[12] García-Salido A, Serrano-González A, Casado-Flores J, Sierra-Colomina M, de Azagra-Garde AM, García-Teresa M, Melen GJ, Ramírez-Orellana M (2018). CD64 on monocytes and granulocytes in severe acute bronchiolitis: Pilot study on its usefulness as a bacterial infection biomarker. J Leukoc Biol.

[13] Vujaklija Brajković A, Košuta I, Tomek D, Rora M, Babel J, Rogić D, Lončar Vrančić A, Radonić R (2021). Utility of procalcitonin in a medical intensive care unit in Croatia. Wien Klin Wochenschr.

[14] Volanakis JE (2001). Human C-reactive protein: expression, structure, and function. Mol Immunol.

[15] Escadafal C, Incardona S, Fernandez-Carballo BL, Dittrich S (2020). The good and the bad: using C reactive protein to distinguish bacterial from non-bacterial infection among febrile patients in low-resource settings. BMJ Glob Health.

[16] Lubell Y, Blacksell SD, Dunachie S, Tanganuchitcharnchai A, Althaus T, Watthanaworawit W, Paris DH, Mayxay M, Peto TJ, Dondorp AM (2015). Performance of C-reactive protein and procalcitonin to distinguish viral from bacterial and malarial causes of fever in Southeast Asia. BMC Infect Dis.

[17] Ansar W, Ghosh S (2013). C-reactive protein and the biology of disease. Immunol Res.

[18] Chauhan N, Tiwari S, Jain U (2017). Potential biomarkers for effective screening of neonatal sepsis infections: an overview. Microb Pathog.

[19] Prkno A, Wacker C, Brunkhorst FM, Schlattmann P (2013). Procalcitonin-guided therapy in intensive care unit patients with severe sepsis and septic shock–a systematic review and meta-analysis. Crit Care.

[20] Paudel R, Dogra P, Montgomery-Yates AA, Coz Yataco A (2020). Procalcitonin: a promising tool or just another overhyped test?. Int J Med Sci.

[21] Hoffmann JJ. Neutrophil CD64: a diagnostic marker for infection and sepsis. Clin Chem Lab Med 47(8):903–16. 10.1515/CCLM.2009.224 19642859

[22] Bhandari V, Wang C, Rinder C, Rinder H (2008). Hematologic profile of sepsis in neonates: neutrophil CD64 as a diagnostic marker. Pediatrics.

[23] Groselj-Grenc M, Ihan A, Derganc M (2008). Neutrophil and monocyte CD64 and CD163 expression in critically ill neonates and children with sepsis: comparison of fluorescence intensities and calculated indexes. Mediators Inflamm.

[24] Dang Y, Lou J, Yan Y, Yu Y, Chen M, Sun G, Li N (2016). The role of the neutrophil Fcγ receptor I (CD64) index in diagnosing spontaneous bacterial peritonitis in cirrhotic patients. Int J Infect Dis.

[25] Xiong SD, Pu LF, Wang HP, Hu LH, Ding YY, Li MM, Yang DD, Zhang C, Xie JX, Zhai ZM (2017). Neutrophil CD64 Index as a superior biomarker for early diagnosis of infection in febrile patients in the hematology department. Clin Chem Lab Med.

[26] Ye Z, Zou H, Liu S, Mei C, Chang X, Hu Z, Yang H, Wu Y (2019). Diagnostic performance of neutrophil CD64 index in patients with sepsis in the intensive care unit. J Int Med Res.

[27] Patnaik R, Azim A, Agarwal V (2020). Neutrophil CD64 a diagnostic and prognostic marker of sepsis in adult critically ill patients: A brief review. Indian J Crit Care Med.

[28] Hung SK, Lan HM, Han ST, Wu CC, Chen KF (2020). Current evidence and limitation of biomarkers for detecting sepsis and systemic infection. Biomedicines.

[29] Wang X, Li ZY, Zeng L, Zhang AQ, Pan W, Gu W, Jiang JX (2015). Neutrophil CD64 expression as a diagnostic marker for sepsis in adult patients: a meta-analysis. Crit Care.

[30] Muzlovic I, Ihan A, Stubljar D (2016). CD64 index on neutrophils can diagnose sepsis and predict 30-day survival in subjects after ventilator-associated pneumonia. J Infect Dev Ctries.

[31] Fontela PS, O’Donnell S, Papenburg J (2018). Can biomarkers improve the rational use of antibiotics?. Curr Opin Infect Dis.

[32] Dimoula A, Pradier O, Kassengera Z, Dalcomune D, Turkan H, Vincent JL (2014). Serial determinations of neutrophil CD64 expression for the diagnosis and monitoring of sepsis in critically ill patients. Clin Infect Dis.

[33] Liu Q, Gao Y, Ou Q, Xu Y, Zhou Z, Li T, Lu Y, Sun F, Zhou X, Li Y (2020). Differential expression of CD64 in patients with Mycobacterium tuberculosis infection: A potential biomarker for clinical diagnosis and prognosis. J Cell Mol Med.

[34] Ryan T, Coakley JD, Martin-Loeches I (2017). Defects in innate and adaptive immunity in patients with sepsis and health care associated infection. Ann Transl Med.

[35] Hohlstein P, Gussen H, Bartneck M, Warzecha KT, Roderburg C, Buendgens L, Trautwein C, Koch A, Tacke F (2019). Prognostic relevance of altered lymphocyte subpopulations in critical illness and sepsis. J Clin Med.

[36] He W, Xiao K, Fang M, Xie L (2021). Immune cell number, phenotype, and function in the elderly with sepsis. Aging Dis.

