# Supplementary material for: Neutrophil CD64 index as a superior indicator for diagnosing, monitoring bacterial infection, and evaluating antibiotic therapy: a case control study
Source: BMC Infect Dis. 2022 Nov 28;22:892. doi: 10.1186/s12879-022-07725-4 (PMC9703738; doi:10.1186/s12879-022-07725-4)
Supplement: Supplementary file 1 — Additional file 1: Figure S1. Comparison of indicators in patients with negative bacterial culture beforeand after appropriate antibiotic therapy. [file 12879_2022_7725_MOESM1_ESM.pptx]

## Slide 1
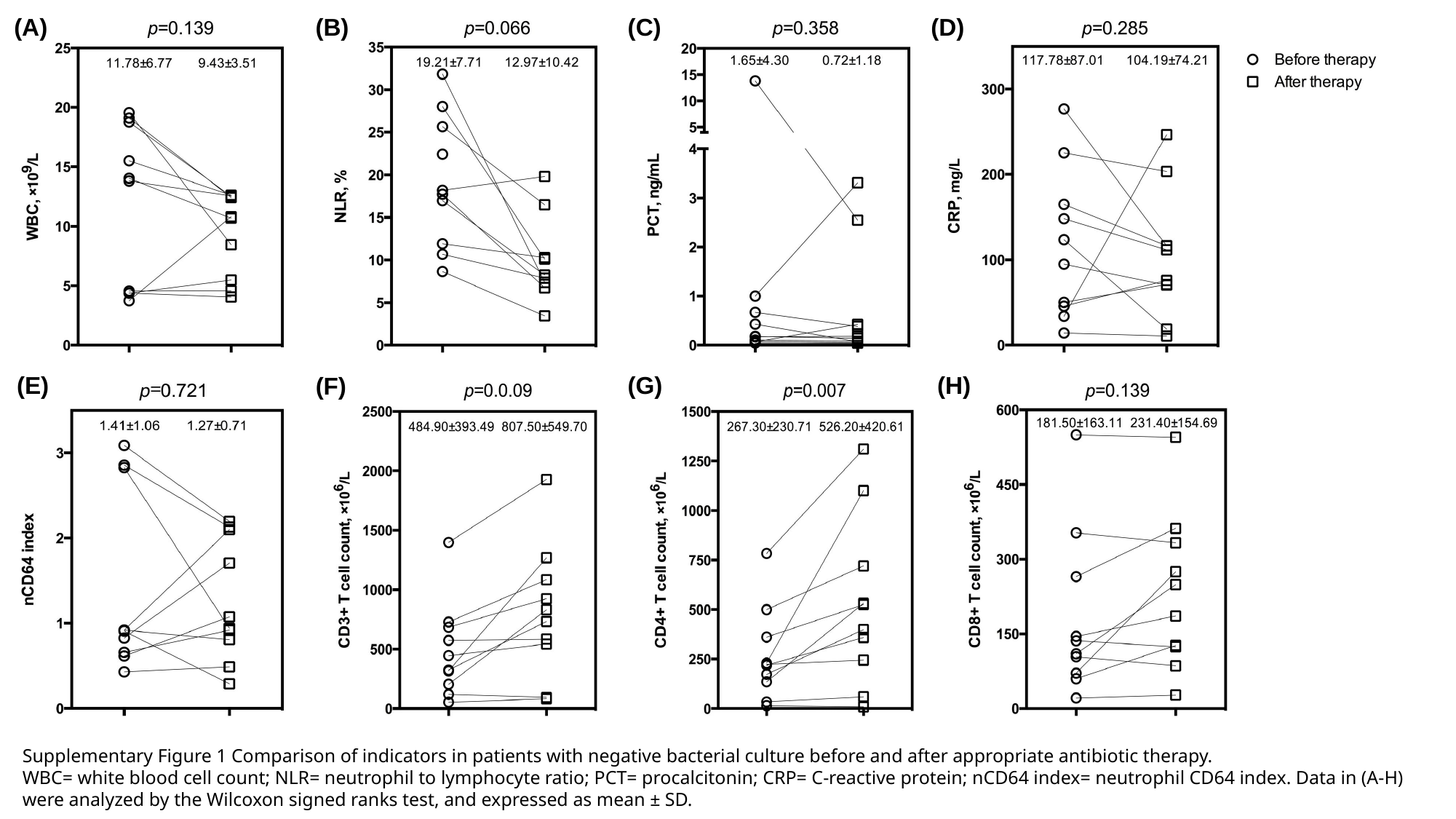

(A)
(B)
(C)
(D)
(E)
(G)
(H)
(F)
Supplementary Figure 1 Comparison of indicators in patients with negative bacterial culture before and after appropriate antibiotic therapy.
WBC= white blood cell count; NLR= neutrophil to lymphocyte ratio; PCT= procalcitonin; CRP= C-reactive protein; nCD64 index= neutrophil CD64 index. Data in (A-H) were analyzed by the Wilcoxon signed ranks test, and expressed as mean ± SD.
