# Supplementary material for: Neutrophil CD64 index as a superior indicator for diagnosing, monitoring bacterial infection, and evaluating antibiotic therapy: a case control study
Source: BMC Infect Dis. 2022 Nov 28;22:892. doi: 10.1186/s12879-022-07725-4 (PMC9703738; doi:10.1186/s12879-022-07725-4)
Supplement: Supplementary file 2 — Additional file 2: Figure S2. The daily alteration of the nCD64 index, PCT, and temperature in infectedpatients. [file 12879_2022_7725_MOESM2_ESM.pptx]

## Slide 1
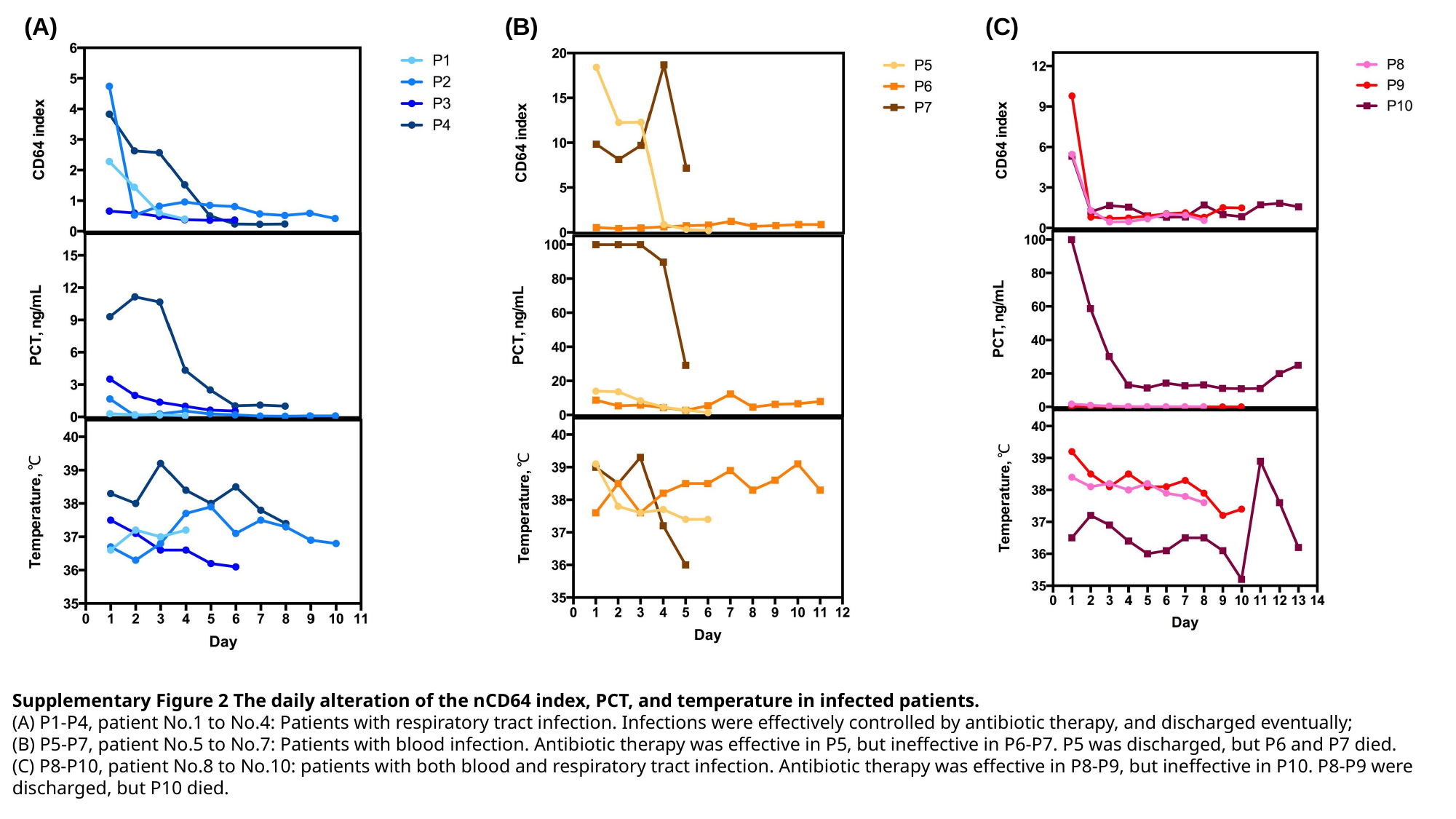

(A)
(B)
(C)
Supplementary Figure 2 The daily alteration of the nCD64 index, PCT, and temperature in infected patients.
(A) P1-P4, patient No.1 to No.4: Patients with respiratory tract infection. Infections were effectively controlled by antibiotic therapy, and discharged eventually;
(B) P5-P7, patient No.5 to No.7: Patients with blood infection. Antibiotic therapy was effective in P5, but ineffective in P6-P7. P5 was discharged, but P6 and P7 died.
(C) P8-P10, patient No.8 to No.10: patients with both blood and respiratory tract infection. Antibiotic therapy was effective in P8-P9, but ineffective in P10. P8-P9 were discharged, but P10 died.
