# Supplementary material for: Neutrophil CD64 index as a superior indicator for diagnosing, monitoring bacterial infection, and evaluating antibiotic therapy: a case control study
Source: BMC Infect Dis. 2022 Nov 28;22:892. doi: 10.1186/s12879-022-07725-4 (PMC9703738; doi:10.1186/s12879-022-07725-4)
Supplement: Supplementary file 3 — Additional file 3: Table S1. The distributionof bacteria strains. [file 12879_2022_7725_MOESM3_ESM.docx]

**Additional file 3: Table S1. The distribution of bacteria strains**

| **Respiratory Tract Infection Group (n=45)** | | **Blood Infection Group (n=27)** | | | |
| --- | --- | --- | --- | --- | --- |
|  |  | **Simple Blood Infection Subgroup (n=16)** | | **Mixed Blood Infection Subgroup (n=11)** | |
| **Bacteria strains of respiratory tract infection** | | **Bacteria strains of blood infection** | | **Bacteria strains of blood infection** | |
| Acinetobacter baumannii | 9(20.00%) | Acinetobacter baumannii | 1(6.25%) | Acinetobacter baumannii | 1(9.09%) |
| Escherichia coli | 4(8.89%) | Acinetobacter johnsonii | 1(6.25%) | Klebsiella pneumoniae | 2(18.18%) |
| Klebsiella pneumoniae | 8(17.78%) | Escherichia coli | 2(12.50%) | Escherichia coli | 1(9.09%) |
| Klebsiella aerogenes | 1(2.22%) | Enterobacter aerogenes | 1(6.25%) | Staphylococcus aureus | 1(9.09%) |
| Pseudomonas aeruginosa | 6(13.33%) | Bacteroides thetaiotaomicro | 1(6.25%) | Staphylococcus epidermidis | 1(9.09%) |
| Stenotrophomonas maltophilia | 1(2.22%) | Staphylococcus aureus | 2(12.50%) | Staphylococcus hominis | 1(9.09%) |
| Enterobacter cloacae | 1(2.22%) | Staphylococcus epidermidis | 1(6.25%) | Two or more bacteria  mixed infection | 4(36.36%) |
| Staphylococcus aureus | 8(17.78%) | Staphylococcus hominis | 1(6.25%) |  |  |
| Two or more bacteria  mixed infection | 7(15.56%) | Staphylococcus haemolyticus | 1(6.25%) |  | |
|  |  | Staphylococcus warneri | 1(6.25%) | **Bacteria strains of respiratory tract infection** | |
|  |  | Gemella mobillorum | 1(6.25%) | Klebsiella pneumoniae | 3(27.27%) |
|  |  | Enterococcus faecium | 2(12.50%) | Pseudomonas aeruginosa | 2(18.18%) |
|  |  | Two or more bacteria  mixed infection | 1(6.25%) | Staphylococcus aureus | 3(27.27%) |
|  |  |  |  | Enterococcus faecium | 1(9.09%) |
|  |  |  |  | Two or more bacteria  mixed infection | 2(18.18%) |
|  |  |  |  |  |  |

Data were presented as n(%).
